# Exploration of the value of progesterone and progesterone/estradiol ratio on the hCG trigger day in predicting pregnancy outcomes of PCOS patients undergoing IVF/ICSI: a retrospective cohort study

**DOI:** 10.1186/s12958-021-00862-6

**Published:** 2021-12-11

**Authors:** Yiqing Yang, Bowen Liu, Gengxiang Wu, Jing Yang

**Affiliations:** 1 Reproductive Medical Centre, Renmin Hospital of Wuhan University, Wuhan, 430060 People’s Republic of China; 2 Hubei Clinical Research Center for Assisted Reproductive Technology and Embryonic Development, Wuhan, 430060 People’s Republic of China

**Keywords:** Progesterone, Progesterone/estradiol, PCOS, Pregnancy, Human chorionic gonadotropin

## Abstract

**Background:**

Polycystic ovary syndrome (PCOS) is a common endocrine disorder with the disorders of estrogen(E2) and progesterone(P) secretion. The purpose of this study was to evaluate the association between the progesterone level or progesterone/estradiol(P/E2) ratio on human chorionic gonadotropin (hCG) trigger day and the outcome of in vitro fertilization in PCOS patients and explore the value of progesterone and P/E2 ratio for predicting the clinical pregnancy.

**Methods:**

The clinical data of 1254 PCOS patients who satisfied the inclusion criteria were retrospectively analyzed, including baseline characteristics such as age, body mass index, basal sex hormone levels, et al., as well as ovarian stimulation data and clinic outcome.

**Results:**

The number of follicles larger than 14 mm in diameter (*P *< 0.001) and retrieved oocytes (*P *< 0.001) was greater in the high progesterone group (progesterone ≥ 0.92 ng/mL). In the high P/E2 group(P/E2 ratio ≥ 0.3), the number of follicles larger than 14 mm in diameter (*P *< 0.001) and retrieved oocytes (*P *< 0.001), as well as the rate of high-quality embryos (*P* = 0.040) were significantly decreased. In ultralong GnRH agonist protocol, the implantation rate(*P *< 0.001), hCG positive rate (*P *< 0.001), clinical pregnancy rate (*P *< 0.001) and live birth rate (*P *< 0.001) were all significantly higher than long GnRH agonist protocol and GnRH antagonist protocol. The clinical pregnancy rate of high progesterone group was significantly lower than that of low progesterone group in ultralong GnRH agonist (*P* = 0.008). The progesterone level could be used as an indicator to predict the positive clinical pregnancy (long GnRH agonist: *P* = 0.001; ultralong GnRH agonist: *P* < 0.001) except in cycles using GnRH antagonist (*P* = 0.169). In the ultralong GnRH agonist, the value of progesterone level in the prediction of clinical pregnancy was significantly higher than that of the P/E2 ratio (*P* = 0.021).

**Conclusions:**

In PCOS patients, the progesterone level is associated with clinical pregnancy rate while P/E2 ratio is not. In subgroup analysis using three different COS protocols, a significant association between progesterone level and clinical pregnancy rate can be observed in the long GnRH agonist protocol and ultralong GnRH agonist protocol. The progesterone level is significantly better than the P/E2 ratio in predicting the pregnancy outcome of PCOS patients, especially in ultralong GnRH agonist cycles.

**Supplementary Information:**

The online version contains supplementary material available at 10.1186/s12958-021-00862-6.

## Introduction

Polycystic ovary syndrome (PCOS) is a common endocrine disorder, affecting approximately 6–25% of women of childbearing age worldwide [1]. Studies have shown that PCOS is the main cause of ovulatory dysfunction infertility, accounting for about 80% of anovulatory infertility [2]. PCOS is a heterogeneous syndrome with various clinical phenotypes, including hyperandrogenemia, menstrual disorder and polycystic ovary morphology. In addition to abnormal reproductive function and endocrine disorder, PCOS is also a high-risk factor for cardiovascular disease, type 2 diabetes and endometrial cancer [3].

In vitro fertilization (IVF) is an important method to treat anovulatory infertility and improve the pregnancy rate of women with PCOS. It has been reported that women with polycystic ovary syndrome are at a higher risk of adverse pregnancy outcomes including miscarriage and very preterm delivery after adjusting for differences in maternal characteristics [4]. Another study also proposed that compared with normal individuals, PCOS patients have a series of adverse pregnancy outcomes, such as low embryo implantation rate and high miscarriage rate. This may be attributable to decreased endometrial receptivity. Abnormal levels of sex hormones and their receptors act an important role in endometrial receptivity impairment in PCOS patients [5]. Even with the same ovulation stimulation protocol, PCOS patients can produce more than 3 times of follicles than non-PCOS patients. These women are more sensitive to gonadotropins(Gn) and even respond to the endogenous release of Gn [6]. As been reported, women with high responsiveness to Gn have a low embryo implantation rate [7].

A consensus has emerged that improving endometrial receptivity is the key factor to improve the pregnancy rate. Estradiol(E2) plays a major role in the initial differentiation of the endometrium. Progesterone(P) promotes the transformation of the endometrium from the proliferative stage to the secretory stage, making the endometrium suitable for embryo implantation and growth. In spite of the routine suppression of the endogenous Gn during controlled ovarian stimulation (COS), elevated progesterone has been observed in cases. The prematurely elevated progesterone level advances the receptivity window of the endometrium, leading to asynchrony between the endometrium and embryo [8], causing implantation failure and affecting the pregnancy outcomes [9]. There are many reasons accounting for the increased serum progesterone level on the human chorionic gonadotropin (hCG) trigger day. The level of progesterone in peripheral blood is directly related to the number of follicles and dosage of exogenous Gn [8]. Additionally, the type of gonadotropin may also have a certain impact on progesterone. Andersen et al. [10] and Bosch et al. [11] studied long GnRH agonist and GnRH antagonist protocols, respectively, and found that the progesterone level in the protocols using recombinant FSH (r-FSH) was higher than that of high-purity human menopausal gonadotropin (HMG).

There is an ongoing debate regarding whether the levels of E2 and progesterone on the hCG trigger day are related to the outcome of pregnancy. It has been found that premature luteinization that is defined as an increased serum progesterone level above a certain threshold on the day of hCG administration following an undesirable early rise in luteinizing hormone (LH) in the follicular phase in IVF embryo transfer cycle is related to a lower pregnancy rate and implantation rate [12]. Another research showed that patients with elevated progesterone and estradiol levels had lower clinical pregnancy rates and live birth rates but higher ectopic pregnancy rates on the day of hCG administration [13]. However, a series of studies found that no association between single E2 concentrations and achievement of pregnancy [13, 14]. Due to the different cutoff values, different patient characteristics and different ovarian stimulation protocols, the results of a series of researches contradicted with each other [15]. The secretion of progesterone and E2 in PCOS patients is different from that in normal population. Considering the persistent ovulation disorder and impaired luteal development in PCOS patients, the progesterone level decreased compared with non-PCOS patients [5]. In the meanwhile, estrogen receptor α was further activated after PCOS patients entered the secretory phase, thus the E2 level was upregulated. As a result, long-term elevated E2 and decreased progesterone levels in the secretory phase of the endometrium from women with PCOS impaired endometrial receptivity [16].. Dominant follicles produce large amounts of estradiol but PCOS follicles do not as they are arrested at the antral follicle stage. In PCOS, aromatase expression and thus E2 synthesis in large antral follicles is impaired [17].

The increased progesterone may be the main negative factor of pregnancy outcome. When combined with elevated progesterone, a high E2 concentration may have a potentially detrimental effect on pregnancy outcomes [13]. A study concluded that the P/E2 ratio was a better predictor than serum progesterone alone in predicting pregnancy outcomes [18]. However, there was conflicting evidence regarding whether the P/E2 ratio was related to the IVF outcomes [15, 19, 20]. At present, few studies have investigated the prognostic effect of the P/E2 ratio on the hCG trigger day in IVF and intracytoplasmic sperm injection (ICSI) cycles. Most of these studies were conducted in long GnRH agonist protocols and evaluated the association between the P/E2 ratio and pregnancy outcomes using arbitrary thresholds [21, 22]. So far, no research has been performed to explore the effect of P/E2 ratio on IVF outcome of PCOS patients. Considering that PCOS patients usually show different endocrine characteristics and ovary response from other infertile patients, previous results regarding the effect of progesterone and P/E2 ratio on IVF outcome may not suitable for PCOS patients.

To the best of our knowledge, investigations regarding the association between the progesterone levels or P/E2 ratio on the hCG trigger day and pregnancy outcomes are scarce regarding the PCOS population. In this study, the clinical data of 1254 patients who had undergone IVF/ICSI cycles from June 2013 to September 2020 were retrospectively analyzed to explore the relationship between serum progesterone and P/E2 ratio on the day of hCG trigger and pregnancy outcomes in PCOS.

## Materials and methods

### Patients and study design

PCOS patients who underwent IVF/ICSI-embryo transfer (ET) between June 2013 and September 2020 at Reproductive Medicine Center of Renmin Hospital of Wuhan University were evaluated. Inclusion criteria: (1) patients with PCOS were diagnosed according to Rotterdam diagnostic criteria [23]; (2) IVF or ICSI was used for insemination; (3) only the first IVF/ICSI-ET cycle was included; (4) COS protocols were long Gonadotropin releasing hormone (GnRH) agonist, ultralong GnRH agonist and GnRH antagonist. Exclusion criteria: (1) patients with infertility caused by other factors; (2) Donor cycles; (3) patients with recurrent spontaneous abortion; (4) One of the couples had chromosome abnormality; (5) Preimplantation genetic testing (PGT) was performed. To determine the cutoff values of the progesterone level and P/E2 ratio to discriminate between positive and negative clinical pregnancy outcomes, a receiver operating curve (ROC) analysis was performed. The calculation of the P/E2 ratio is equal to progesterone (ng/ml) × 1000/estradiol (pg/ml). Patients were divided into two groups according to serum progesterone level (progesterone < 0.92 ng/ml and progesterone ≥ 0.92 ng/ml) and P/E2 ratio (P/E2 ratio < 0.3, P/E2 ratio ≥ 0.3) based on cutoffs determined by ROC analysis. Besides, patients were further divided into three groups (long GnRH agonist, ultralong GnRH agonist and GnRH antagonist) according to different ovarian stimulation protocols.

### Protocols for ovarian stimulation

#### Long GnRH agonist protocol

The patient started to take one tablet of oral contraceptive pills every day (Diane-35, Bayer, Germany) from the third day of the menstrual cycle, and subcutaneous injection of 0.1 mg/d of short-acting GnRH agonist (GnRH-a, triptorelin, Ferring, Germany) was started on the 19th day for downregulation of pituitary. After 14 days of continuous injection, transvaginal ultrasound and sex hormone levels were examined to determine whether the condition reached the downregulation standard (FSH < 5 U/L, LH < 5 U/L, E2 < 50 pg/ml, endometrial thickness < 5 mm and follicle diameter < 5 mm). If the patients satisfied the criteria of downregulation, GnRH-a was changed to 0.05 mg/day and continued until the trigger day, and recombinant human follicle stimulating hormone (r-FSH, Merck Serono, Switzerland) or human menopausal gonadotropin (hMG, Livzon, China) was injected subcutaneously or intramuscularly every day. The starting dosage of r-FSH was 100–200 U/d according to the ovarian response**.** Transvaginal ultrasound monitoring and sex hormone level determination were performed after 3–4 days of continuous injection. The dosage was adjusted according to the ovarian response. When the diameters of 2 follicles were more than 18 mm or 3 follicles were more than 17 mm, 6000–10000 IU of human chorionic gonadotropin (hCG, Livzon, China) was administered intramuscularly for triggering. Oocyte aspiration was performed approximately 34–36h after triggering by a standard transvaginal ultrasound-guided approach. One to two embryos were transferred 3–5 days after oocyte aspiration.

#### Ultralong GnRH agonist protocol

3.75 mg of long-acting GnRH-a (Leuprorelin Acetate, China) was injected intramuscularly on the day 2–3 of menstrual cycle. Ultrasound and sex hormone examinations were performed 28 days later. If the follicles and hormones satisfied the downregulation standard prescribed before, ovarian stimulation was started, the starting dose of Gn, monitoring process, Gn adjustment, trigger, oocyte aspiration and embryo transfer strategy were as the same as the long GnRH agonist protocol.

#### GnRH antagonist protocol

Gn was injected every day from the second day of menstruation. When the diameter of the follicle was larger than 14 mm, 0.25 mg of the antagonist (Merck Serono, Germany) was applied until the trigger day. The starting dose of Gn, monitoring process, Gn adjustment, trigger, oocyte aspiration and embryo transfer strategy were as the same as the long GnRH agonist protocol.

Experienced clinicians determine the patient’s COS protocol according to different condition. Long agonist was generally used for patients with relatively fewer AFCs. Ultralong agonist protocol and antagonist protocol were used for people at a higher risk of developing ovarian hyperstimulation syndrome (OHSS).

### Freeze-all

Indications for the freeze-all policy included a high risk of developing OHSS (women who are younger than 35 years old, use hCG for ovarian stimulation or have a high response to Gn, the number of follicles > 14 mm is more than 20 or E2 level is more than 5000 pg/ml on trigger day), inadequate endometrial thickness (< 7 mm), high progesterone level(> 2.0 ng/ml) and some other conditions (such as high blood pressure, fever, individual preference).

### Cycle cancellation

If there were no oocytes retrieved or no transplantable embryo, the cycle would be cancelled.

### Pregnancy diagnosis

According to the Peter cleavage embryo scoring system, the embryos were divided into grades I-IV according to the size, shape and fragmentation rate of blastomeres. Grade I and II embryos were defined as high-quality embryos. On the 3rd to 5th day after oocyte retrieval, high-quality embryos or blastocysts were selected for embryo transfer. The hCG level in peripheral blood was measured on the 14th day after embryo transfer. Clinical pregnancy was defined as pregnancy with positive fetal cardiac activity in the 6th week±1/2 weeks of gestation by transvaginal ultrasound. Early miscarriage was defined as embryo loss before 12 weeks of pregnancy.

### Outcome assessment

The primary outcomes were the clinical pregnancy rate (CPR) and live birth rate (LBR). CPR was defined as (the number of cycles with clinical pregnancy/the number of all cycles of embryo transfer*100%). LBR was defined as (the number of live birth cycles/the number of all cycles of embryo transfer*100%). The secondary outcome was the cycle cancellation rate, embryo freeze-all rate, implantation rate and early miscarriage rate. The implantation rate was defined as (the number of total gestational sacs/the number of total transferred embryos*100%). The early miscarriage rate was defined as (the number of cycles with embryo loss before 12 weeks of gestation/the total number of all cycles of embryo transfer*100%).

### Statistical analysis

SPSS 19.0 software was used for statistical analysis. ROC analysis was performed to identify the most effective progesterone and P/E2 cutoff values, that can discriminate between successful and unsuccessful pregnancy outcomes for PCOS women. The optimal cutoff value was determined according to the equivalent sensitivity and specificity and maximum value of the area under the ROC curve. Youden index (sensitivity + specificity-1) was used to choose the cutoff points of progesterone and P/E2 ratio. All continuous variables were expressed as mean ± SD. One-way ANOVA was used to compare the measurement data with a normal distribution between groups, and chi-squared test was used to compare the count data between groups. When significant differences among the three groups were observed, pairwise comparisons using One-way ANOVA were further conducted. A multivariate logistic regression model was also used to examine the preferential effects of progesterone and P/E2 ratio on the clinical pregnancy rates. Age, body mass index (BMI), Infertility duration, antral follicle count (AFC), fasting serum glucose level, stimulation type, basal FSH, basal LH, basal E2 were included in multivariable regression model. ROC curves were constructed to examine the diagnostic value of progesterone and P/E2 ratio and the area under the curve (AUC) was computed. The AUC represents the probability of correctly predict the clinical pregnancy of the controls and patients with PCOS. *P* < 0.05 indicates that the difference is statistically significant**.**

## Results

**Fig. 1:**
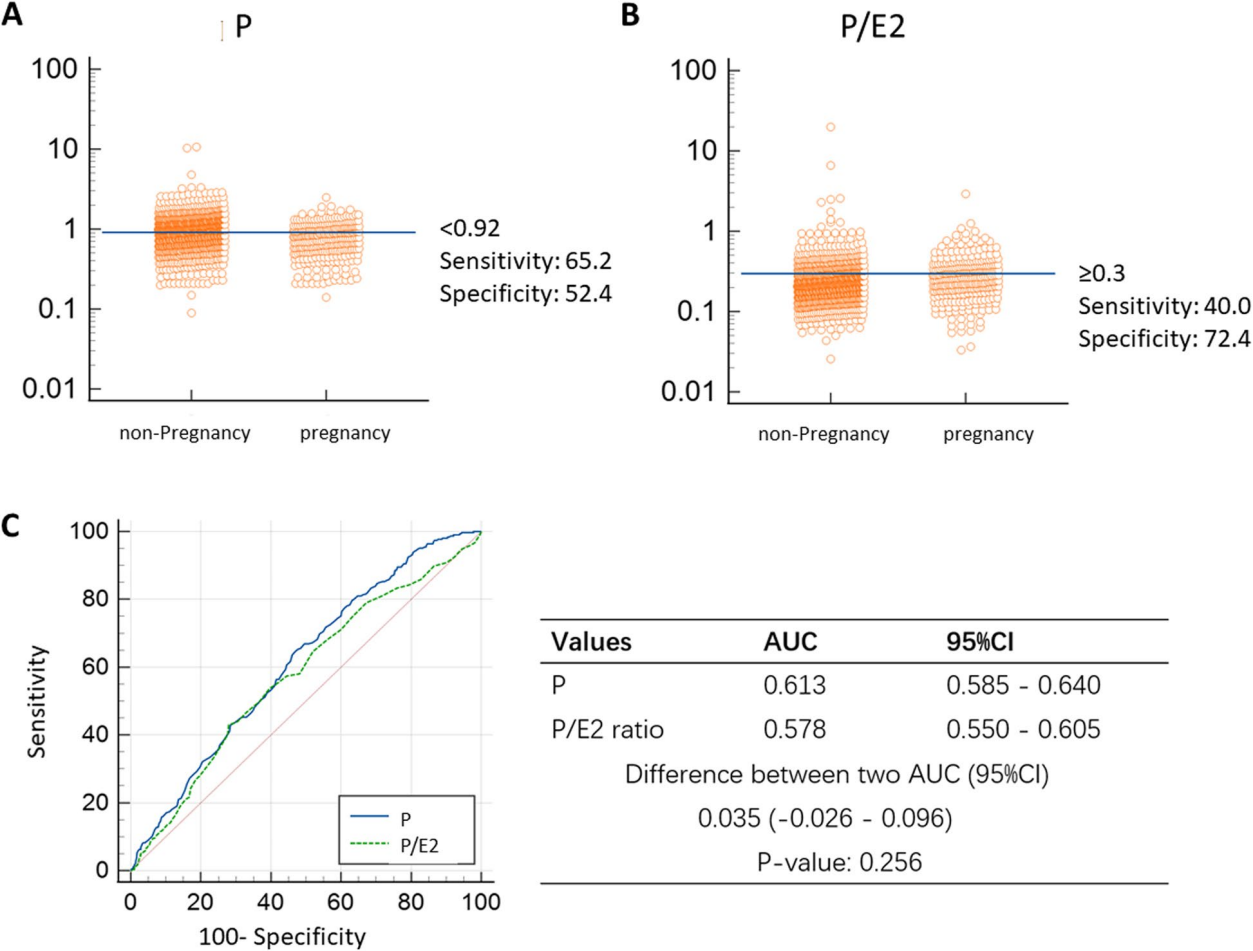
ROC curve. **A**: Interactive dot diagram of P level. The cutoff of P level is 0.92. **B**: Interactive dot diagram of P/E2 ratio. The cutoff of P/E2 ratio is 0.3. **C**: Comparison of ROC curves between P and P/E2 ratio based on clinical pregnancy. AUC: area under the ROC curve. CI: confidence interval

A total of 1254 women were enrolled. The baseline characteristics and ovarian stimulation data of these women with pregnancy and non-pregnancy were shown in Table 1. Compared with the non-pregnancy group, women with clinical pregnancy were younger (*P* = 0.016) and had less AFC (*P* = 0.001), less matured follicles (*P* < 0.001), and lower basal LH levels (*P* = 0.021). Days of stimulation of women with clinical pregnancy were longer (*P* = 0.008) and Gn dosages were more (*P* = 0.017). Moreover, the progesterone levels on hCG trigger day were lower than the women with non-pregnancy (*P* < 0.001), but the P/E2 ratio was of no significant difference between the two groups (*P* > 0.05). ROC results were presented in Fig. 1. The cutoff points of P and P/E2 ratio were selected by the Youden index. The cutoff of progesterone was 0.92 ng/mL (Fig. 1A) and the cutoff of P/E2 is 0.3 (Fig. 1B). AUC of the progesterone level and P/E2 ratio on the hCG trigger day for predicting the clinical pregnancy outcome were 0.613[95%CI (0.585–0.640), *P *< 0.001] and 0.578[95%CI (0.541–0.615), *P* < 0.001], respectively.

**Table 1:** The baseline characteristics and ovarian stimulation data of case (women with clinical pregnancy) and control groups

	Clinical Pregnancy	No Clinical Pregnancy	*P*-value
Variables	*N* = 305	*N* = 949	
Age (years)	28.8 ± 3.4	29.4 ± 3.6	**0.016**
BMI^a^ (kg/m^2^)	23.8 ± 3.3	25.3 ± 26.2	0.307
Infertility duration (years)	4.1 ± 2.6	4.1 ± 2.6	0.644
AFC^b^	21.4 ± 8.7	23.5 ± 9.9	**0.001**
Basal FSH^c^ (IU/L)	6.5 ± 2.0	6.4 ± 2.6	0.826
Basal LH^d^ (IU/L)	6.2 ± 5.2	7.0 ± 5.1	**0.021**
Basal estrogen (pg/mL)	49.6 ± 57.1	59.1 ± 166.7	0.338
Fasting serum glucose (mmol/L)	5.1 ± 0.7	5.1 ± 0.6	0.271
Follicles≥14 mm^*^	14,0 ± 5.3	17.8 ± 7.6	**< 0.001**
Days of stimulation	11.3 ± 3.3	10.8 ± 3.1	**0.008**
Gn dosage^e^ (IU/L)	2577.5 ± 1237.6	2401.2 ± 1078.4	**0.017**
Serum estrogen (pg/mL) ^*^	3302.2 ± 2593.0	4728.2 ± 2755.5	**< 0.001**
Serum Progesterone (ng/mL) ^*^	0.8 ± 0.4	1.0 ± 0.7	**< 0.001**
Serum P/E2 ratio ^*^	0.3 ± 0.2	0.3 ± 0.7	0.765
Endometrial thickness on ET day (cm)	1.1 ± 0.3	1.0 ± 0.2	**< 0.001**
Oocytes retrieved	12.4 ± 4.7	16.7 ± 7.9	**< 0.001**

^*^Measured on the day of hCG

^a^BMI: body mass index

^b^AFC: antral follicle count

^c^FSH: follicle-stimulating hormone

^d^LH: luteinizing hormone

^e^Gn: gonadotropin

At an optimum cutoff of 0.92 ng/mL, the sensitivity and specificity of progesterone were 65.2 and 52.4%, respectively. The P/E2 ratio at the cutoff of 0.3 showed a lower sensitivity but higher specificity (40.0 and 72.4%, respectively). There was no statistically significant difference in the predictive value of progesterone and P/E2 ratio in the prediction of clinical pregnancy, as the difference of AUC between progesterone and P/E2 ratio was 0.035 (95% CI: − 0.026-0.096, *P* = 0.256) (Fig. 1C).

In Table 2, baseline characteristics and pregnancy outcomes with different levels of progesterone and P/E2 ratio were presented. In the high progesterone group (progesterone ≥ 0.92 ng/mL), the number of follicles larger than 14 mm in diameter (*P* < 0.001) and retrieved oocytes (*P* < 0.001) were greater than the low progesterone group (progesterone < 0.92 ng/mL). However, in the high P/E2 ratio group, the number of follicles larger than 14 mm in diameter(*P* < 0.001) and retrieved oocytes (*P *< 0.001), as well as the rate of high-quality embryos (*P* = 0.040) were significantly decreased, but the days of stimulation (*P* = 0.005) and the total dosage of Gn (*P *< 0.001) were higher. The cycle cancellation rate was higher in the high progesterone group but lower in the high P/E2 group (*P *< 0.001). High progesterone and high P/E2 ratio both had no significant effect on CPR and LBR (*P *> 0.05).

**Table 2:** Baseline characteristics and pregnancy outcomes with different levels of P and P/E2 ratio

	Progesterone^a^			Progesterone/estradiol (P/E2) ratio^a^		
	*P* < 0.92 ng/mL	P ≥ 0.92 ng/mL		P/E2 ratio < 0.3	P/E2 ratio ≥ 0.3	
Characteristics	*N* = 642	*N* = 612	*P* value	*N* = 865	*N* = 389	P value
Age (years)	29.1 ± 4.0	29.4 ± 3.6	0.067	29.2 ± 3.5	29.4 ± 3.6	0.262
BMI (kg/m^2^)	25.6 ± 24.0	24.3 ± 21.6	0.292	24.0 ± 16.8	27.0 ± 32.4	**0.036**
Infertility duration (years)	4.2 ± 2.6	4.1 ± 2.6	0.485	4.1 ± 2.6	4.3 ± 2.7	0.209
AFC	23.4 ± 10.0	22.7 ± 9.3	0.200	23.7 ± 9.9	21.5 ± 8.9	**<0.001**
Basal FSH (IU/L)	6.5 ± 1.8	6.4 ± 2.9	0.244	6.5 ± 2.6	6.4 ± 1.9	0.634
Basal LH (IU/L)	6.8 ± 5.3	6.8 ± 4.9	0.965	7.1 ± 5.1	6.0 ± 4.9	**<0.001**
Basal estrogen (pg/mL)	57.2 ± 153.5	56.4 ± 141.7	0.923	61.7 ± 176.9	45.9 ± 28.8	0.083
Fasting serum glucose (mmol/L)	5.1 ± 0.7	5.1 ± 0.6	0.561	5.1 ± 0.6	5.2 ± 0.7	0.129
Follicles≥14 mm^a^	15.2 ± 6.8	18.6 ± 7.3	**<0.001**	17.9 ± 7.2	14.0 ± 6.4	**<0.001**
Days of stimulation	11.3 ± 3.4	10.6 ± 2.8	**<0.001**	10.8 ± 3.2	11.3 ± 3.0	**0.005**
Gn dosage (IU/L)	2511.0 ± 1192.8	2373.9 ± 1037.4	**0.030**	2303.9 ± 1085.9	2755.8 ± 1137.2	**<0.001**
Serum estrogen (pg/mL) ^a^	3378.8 ± 2035.7	5433.1 ± 3063.2	**<0.001**	5189.4 ± 2876.9	2584.5 ± 1380.8	**<0.001**
Serum Progesterone (ng/mL) ^a^	0.6 ± 0.2	1.4 ± 0.7	**<0.001**	0.9 ± 0.4	1.2 ± 0.9	**<0.001**
Serum P/E2 ratio ^a^	0.2 ± 0.2	0.4 ± 0.9	**<0.001**	0.2 ± 0.1	0.5 ± 1.1	**<0.001**
Endometrial thickness on ET day (cm)	1.1 ± 0.3	1.0 ± 0.2	**0.002**	1.0 ± 0.2	1.1 ± 0.2	0.066
Oocytes retrieved	13.1 ± 6.4	18.5 ± 7.4	**<0.001**	17.0 ± 7.4	12.8 ± 6.7	**<0.001**
2PN oocytes rate (%)	55.9% (4670/8350)	56.6% (6398/11301)	0.338	56.3%(8267/14678)	56.3%(2801/4973)	0.998
Cleavage rate of 2PN oocytes (%)	98.0% (4577/4670)	97.6% (6245/6398)	0.159	97.8%(8085/8267)	97.7%(2737/2801)	0.796
High-quality embryo rate (%)	67.4% (3147/4670)	65.3% (4181/6398)	**0.025**	66.7%(5518/8267)	64.6%(1810/2801)	**0.040**
Outcomes						
Number of embryo transfer cycle	411	250		400	261	
Cycle cancellation rate (%)	36.0% (231/642)	59.2% (362/612)	**<0.001**	53.8%(465/865)	32.9%(128/389)	**<0.001**
Rate of cancelled cycles due to inadequate endometrial thickness (%)	11.3% (26/231)	4.1% (15/362)	**0.001**	5.4%(25/465)	12.5%(16/128)	**0.005**
Rate of cancelled cycles due to OHSS prevention (%)	52.4% (121/231)	63.8% (231/362)	**0.006**	64.1%(298/465)	42.2%(54/128)	**<0.001**
Rate of cancelled cycles due to high P level (%)^a^	0% (0/231)	5.5% (20/362)	**<0.001**	1.3%(6/465)	10.9%(14/128)	**<0.001**
Rate of cancelled cycles due to no embryos obtained (%)	5.2% (12/231)	3.6% (13/362)	0.343	3.0%(14/465)	8.6%(11/128)	**0.005**
Rate of cancelled cycles due to no oocytes retrieved (%)	0.4% (1/231)	0.3% (1/362)	0.748	0.2%(1/465)	0.8%(1/128)	0.328
Rate of cancelled cycles due to other reasons (%)	30.7% (71/231)	22.7% (82/362)	**0.028**	26.0%(121/465)	25%(32/128)	0.815
Freeze-all cycles rate (%)	34.0% (218/642)	56.9% (348/612)	**<0.001**	52.0%(450/865)	29.8%(116/389)	**<0.001**
Implantation rate (%)	34.3% (275/802)	28.7% (139/484)	**0.038**	30.3% (237/782)	35.1% (177/504)	0.071
hCG+ rate (%)	61.1% (251/411)	57.2% (143/250)	0.325	57.3% (229/400)	63.2% (165/261)	0.126
Clinical pregnancy rate (%)	47.9% (197/411)	43.2% (108/250)	0.237	44.8% (179/400)	48.3% (126/261)	0.374
Ectopic pregnancy rate (%)	1.7% (7/411)	2.0% (5/250)	0.782	2.0% (8/400)	1.5% (4/261)	0.660
Early miscarriage rate (%)	4.1% (17/411)	2.8% (7/250)	0.373	4.0% (16/400)	3.1% (8/261)	0.530
Live birth rate (%)	15.6% (64/411)	14.0% (35/250)	0.583	15.8% (63/400)	13.8%(36/261)	0.491

^a^Measured on the day of hCG

Significant differences were shown in E2 (*P *< 0.001), progesterone (*P *< 0.001) and P/E2 ratio (*P* = 0.036) on the hCG trigger day among the three groups of different ovarian stimulation protocols (Table 3). The days of stimulation(*P *< 0.001) and total Gn dosage (*P* = 0.001) of the ultralong GnRH agonist protocol were the highest, and the implantation rate(*P *< 0.001), hCG positive rate (*P *< 0.001), clinical pregnancy rate (*P *< 0.001) and live birth rate (*P *< 0.001) were all significantly higher than long GnRH agonist protocol and GnRH antagonist protocol. Besides, no significant difference was found in the ectopic pregnancy rate or early miscarriage rate among the three groups (*P* > 0.05).

**Table 3:** Baseline characteristics and pregnancy outcomes of patients with different COS protocols

	A: long GnRH agonist	B: ultralong GnRH agonist	C: GnRH antagonist	
Characteristics	*N* = 362	*N* = 369	*N* = 523	P value
Age (years)	29.4 ± 3.7	29.2 ± 3.5	29.1 ± 3.5	0.547
BMI (kg/m^2^)	25.2 ± 25.6	25.8 ± 33.3	24.1 ± 4.0	0.525
Infertility duration (years)	4.3 ± 2.9	4.0 ± 2.4	4.1 ± 2.6	0.468
AFC	18.1 ± 6.2	25.6 ± 10.8	24.5 ± 9.5	**<0.001**^**a**^
Basal FSH (IU/L)	6.2 ± 2.5	6.5 ± 1.8	6.5 ± 2.8	0.097
Basal LH (IU/L)	6.0 ± 4.6	7.7 ± 6.3	6.7 ± 4.3	**<0.001**^**b**^
Basal estrogen (pg/mL)	53.1 ± 124.1	48.0 ± 27.5	65.7 ± 203.4	0.184
Fasting serum glucose (mmol/L)	5.0 ± 0.5	5.1 ± 0.5	5.2 ± 0.8	**<0.001**^**c**^
Follicles≥14 mm^*^	14.1 ± 7.8	17.1 ± 6.9	16.6 ± 7.3	0.176
Days of stimulation	10.3 ± 2.3	12.2 ± 3.2	10.5 ± 3.3	**<0.001**^**d**^
Gn dosage (IU/L)	2456.1 ± 946.1	2604.1 ± 1233.9	2322.9 ± 1136.9	**0.001**^**e**^
Serum estrogen (pg/mL) ^*^	4854.8 ± 2710.9	3477.0 ± 2266.0	4691.8 ± 3012.0	**<0.001**^**f**^
Serum Progesterone (ng/mL) ^*^	0.9 ± 0.4	0.9 ± 0.7	1.1 ± 0.7	**<0.001**^**g**^
Serum P/E2 ratio ^*^	0.2 ± 0.2	0.4 ± 1.0	0.3 ± 0.4	**0.036**^**h**^
Endometrial thickness on ET day ^*^ (cm)	1.0 ± 0.2	1.2 ± 0.3	1.0 ± 0.2	**<0.001**^**i**^
Oocytes retrieved	15.4 ± 7.0	15.7 ± 7.1	15.8 ± 8.0	0.721
2PN oocytes rate (%)	57.6% (3214/5581)	54.5% (3158/5792)	56.7% (4696/8278)	**0.003**^**j**^
Cleavage rate of 2PN oocytes (%)	98.1% (3154/3214)	97.2% (3069/3158)	97.9% (4599/4696)	**0.023**^**k**^
High-quality embryo rate (%)	66.2% (2128/3214)	63.4% (2001/3158)	68.1% (3199/4696)	**<0.001**^**l**^
Number of embryo transfer cycle	214	204	243	
Cycle cancellation rate (%)	40.9%(148/362)	44.7%(165/369)	53.5%(280/523)	**0.001**^**m**^
Rate of cancelled cycles due to inadequate endometrial thickness (%)	3.4%(5/148)	5.5%(9/165)	9.6%(27/280)	**0.036**^**n**^
Rate of cancelled cycles due to OHSS prevention (%)	48.0%(71/148)	74.5%(123/165)	56.4%(158/280)	**<0.001**^**o**^
Rate of cancelled cycles due to high P level (%)^*^	4.1%(6/148)	1.8%(3/165)	3.9%(11/280)	0.427
Rate of cancelled cycles due to no embryos obtained (%)	4.1%(6/148)	4.2%(7/165)	4.3%(12/280)	0.471
Rate of cancelled cycles due to no oocytes retrieved (%)	0.7%(1/148)	0.6%(1/165)	0.0%(0/280)	0.405
Rate of cancelled cycles due to other reasons (%)	39.9%(59/148)	13.3%(22/165)	25.7%(72/280)	**<0.001**^**p**^
Freeze-all cycles rate (%)	39.0%(141/362)	42.5%(157/369)	51.2%(268/523)	**0.001**^**q**^
Implantation rate (%)	31.6% (132/418)	45.0% (175/389)	22.3% (107/479)	**<0.001**^**r**^
HCG+ rate (%)	56.5% (121/214)	71.6% (146/204)	52.3% (127/243)	**<0.001**^**s**^
Clinical pregnancy rate (%)	42.5% (91/214)	62.7% (128/204)	35.4% (86/243)	**<0.001**^**t**^
Ectopic pregnancy rate (%)	1.4% (3/214)	1.0% (2/204)	2.9% (7/243)	0.279
Early miscarriage rate (%)	3.3% (7/214)	4.4% (9/204)	3.3% (8/243)	0.773
Live birth rate (%)	10.7%(23/214)	25.5%(52/204)	9.8%(24/243)	**<0.001**^**u**^

^*^Measured on the day of hCG

Pairwise comparisons (when overall *p* values<0.05)

^a^A versus B<0.001, A versus C<0.001

^b^A versus B<0.001, B versus C = 0.022

^c^A versus C = 0.030

^d^A versus B<0.001, B versus C<0.001

^e^B versus C = 0.002

^f^A versus B <0.001, B versus C<0.001

^g^A versus C = 0.009, B versus C<0.001

^h^A versus B>0.05, A versus C>0.05, B versus C>0.05

^i^A versus B = 0.000, A versus C = 0.038, B versus C<0.001

^j^A versus B = 0.001, B versus C = 0.010

^k^A versus B = 0.012, B versus C = 0.031

^l^A versus B = 0.017, B versus C<0.001

^m^A versus C<0.001, B versus C = 0.009

^n^A versus C = 0.019

^o^A versus B <0.001, B versus C<0.001

^P^A versus B <0.001, A versus C = 0.003, B versus C = 0.002

^q^A versus C<0.001, B versus C = 0.010

^r^A versus B <0.001, A versus C = 0.002, B versus C<0.001

^s^A versus B = 0.001, B versus C<0.001

^t^A versus B<0.001, B versus C<0.001

^u^A versus B<0.001, B versus C<0.001

Subgroup analysis of three different ovarian stimulation protocols was carried out based on the progesterone level and P/E2 ratio on the hCG trigger day. The results were presented in supplementary Table 1 and supplementary Table 2. Among the three protocols, the number of oocytes retrieved in the high progesterone group all increased significantly (long GnRH agonist: *P *< 0.001; ultralong GnRH agonist: *P *< 0.001; GnRH antagonist: *P *< 0.001). The cycle cancellation rate and freeze-all rate both increased significantly (long GnRH agonist: *P *< 0.001; ultralong GnRH agonist: *P* = 0.002; GnRH antagonist: *P *< 0.001). The clinical pregnancy rate of the high progesterone group was significantly lower than that of the low progesterone group in the ultralong GnRH agonist(*P* = 0.008) A high P/E2 ratio had no significant effect on the clinical pregnancy rate, ectopic pregnancy rate, early miscarriage rate and live birth rate(*P* > 0.05), but significantly reduced the cycle cancellation rate in all protocols (*P *< 0.001) and significantly increased the implantation rate of the GnRH antagonist protocol (*P* = 0.036). In the low P/E2 ratio group, the total dose of Gn was lower (long GnRH agonist: *P *< 0.001; ultralong GnRH agonist: *P* = 0.021; GnRH antagonist: *P *< 0.001) and the number of oocytes retrieved was higher (long GnRH agonist: *P* < 0.001; ultralong GnRH agonist: *P *< 0.001; GnRH antagonist: *P *< 0.001) in all protocols.

**Fig. 2:**
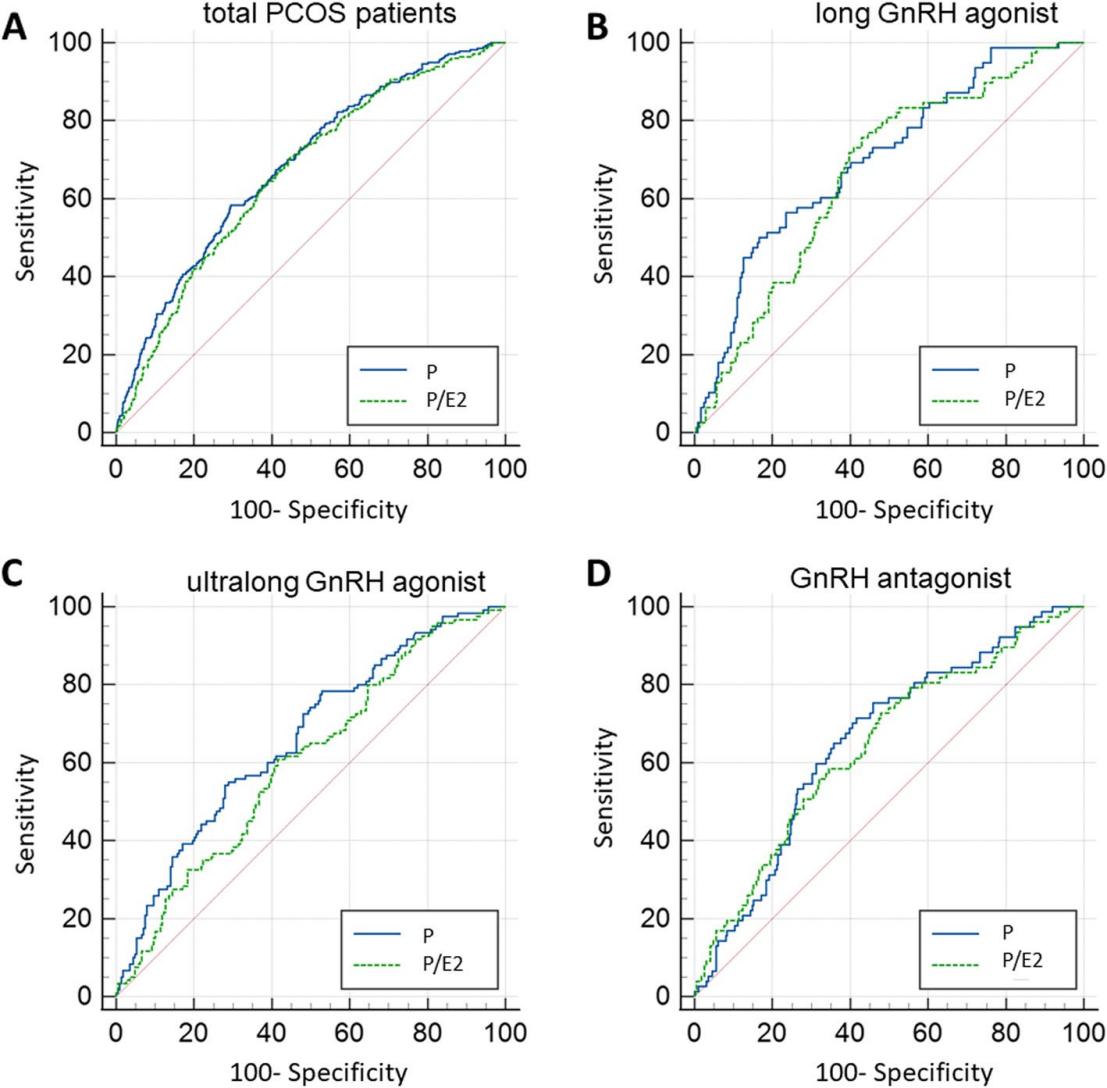
Predicted probability of P and P/E2 ratio in prediction of clinical pregnancy. **A**: Predicted probability of P and P/E2 ratio in prediction of clinical pregnancy of total PCOS patients. **B**: A: Predicted probability of P and P/E2 ratio in prediction of clinical pregnancy of PCOS patients using long GnRH agonist. **C**: A: Predicted probability of P and P/E2 ratio in prediction of clinical pregnancy of PCOS patients using ultralong GnRH agonist. **D**: Predicted probability of P and P/E2 ratio in prediction of clinical pregnancy of PCOS patients using GnRH antagonist

In multivariate logistic regression analysis adjusted for age, BMI, Infertility duration, AFC, fasting serum glucose level, stimulation type, basal FSH, basal LH, basal E2 levels (Table 4). One ng/mL increase in progesterone level was associated with 0.42 times decreased (95% CI: 0.30–0.60; *P* < 0.001) possibility of clinical pregnancy. However, one unit increase in the P/E2 ratio was associated with no significant change in clinical pregnancy (OR 0.97; 95% CI: 0.79–1.20; *P* = 0.774). In three different ovarian stimulation protocols, when considering the influence of other variables, the progesterone level could be used as an indicator to predict the positive clinical pregnancy (long GnRH agonist: *P* = 0.001; ultralong GnRH agonist: *P* < 0.001) except in cycles with GnRH antagonist (*P* = 0.169). Besides, the P/E2 ratio could not be used as an indicator to predict the positive clinical pregnancy in all stimulation types. As shown in Fig. 2, the AUC of progesterone and P/E2 ratio in the prediction of clinical pregnancy were 0.686 and 0.637, respectively. The difference between values of progesterone and P/E2 in the prediction of clinical pregnancy was significant, as the difference between the two AUCs was 0.021[95% CI: 0.002 to 0.040, *P* = 0.034]. In the ultralong GnRH agonist, the value of progesterone level in the prediction of clinical pregnancy was significantly higher than that of the P/E2 ratio (*P* = 0.021). A significant difference between values of progesterone and P/E2 in the prediction of clinical pregnancy was not noted in long GnRH agonist (*P* = 0.158) and GnRH antagonist (*P* = 0.256) (Table 5).

**Table 4:** Association between clinical pregnancy and serum P level or P/E2 ratio on hCG trigger day by multivariable logistic regression analysis

Variables	Adjusted OR	95% CI	p value
**All cycles**			
P(ng/mL)	0.42	0.30–0.60	< 0.001
P/E2 ratio	0.97	0.79–1.20	0.774
**long GnRH agonist**			
P(ng/mL)	0.30	0.14–0.61	0.001
P/E2 ratio	1.44	0.33–6.30	0.626
**ultralong GnRH agonist**			
P(ng/mL)	0.33	0.18–0.60	< 0.001
P/E2 ratio	0.94	0.71–1.24	0.648
**GnRH antagonist**			
P(ng/mL)	0.70	0.41–1.17	0.169
P/E2 ratio	1.14	0.66–1.95	0.638

Age, BMI, Infertility duration, AFC, fasting serum glucose level, stimulation type, basal FSH, basal LH, basal E2 were included in the multivariable regression model

**Table 5:** Predicted probability of P and P/E2 ratio in prediction of clinical pregnancy

Values	AUC	95%CI	Difference between two AUC (95%CI)	P-value
**All cycles**				
P(ng/mL)	0.686	0.658–0.713	0.021(0.002–0.040)	0.034
P/E2 ratio	0.665	0.637–0.692		
**long GnRH agonist**				
P(ng/mL)	0.702	0.649–0.751	0.038(−0.015–0.090)	0.158
P/E2 ratio	0.664	0.610–0.715		
**ultralong GnRH agonist**				
P(ng/mL)	0.662	0.609–0.711	0.059(0.010–0.110)	0.021
P/E2 ratio	0.602	0.549–0.654		
**GnRH antagonist**				
P(ng/mL)	0.658	0.613–0.700	0.013(−0.010–0.035)	0.256
P/E2 ratio	0.645	0.600–0.688		

## Discussion

As far as is known, this was the first study to explore the predictive value of progesterone and P/E2 ratio on the day of hCG administration in pregnancy outcomes in PCOS patients in IVF/ICSI. The study included 1254 PCOS patients from our center. Melo et al. [24] found that the premature elevation of serum progesterone on the day of hCG administration did not appear to have a negative impact on pregnancy outcome in oocyte-donation programs. Li et al. [25] found that when the progesterone level was ≥ 1.25 ng/ml on hCG trigger day, the endometrium was transformed into secretory endometrium in advance, leading to unfavourable effects on pregnancy outcomes. As revealed by electron microscope, the early increase in serum progesterone levels in the late follicular phase of the IVF cycle can lead to premature formation of endometrial pinocytosis, closing the implantation window in advance and reducing the embryo implantation rate [26]. This finding was consistent with ours. The increase in serum progesterone levels on the hCG trigger day led to significant changes in gene expression in the endometrium [27]. Multivariate logistic regression analysis in our study showed that the progesterone level rather than P/E2 ratio can be used to predict clinical pregnancy in PCOS patients after adjusting for the main influencing factors, indicating that the predictive value of P/E2 ratio on the hCG trigger day on pregnancy outcomes in PCOS patients was limited. Another study found that the increased serum progesterone in patients with PCOS on the hCG trigger day was related to a high pregnancy rate [28], which was contradictory to our results. In the meanwhile, scholars also observed that when the serum progesterone was higher than the threshold concentration on the day of hCG administration, the pregnancy outcomes were not affected [29]. In our study, while an elevated progesterone level on the day of ovulation trigger was associated with lower clinical pregnancy rates among all included cycles, the subanalysis stratified by stimulation protocol only confirmed this in the long GnRH agonist and ultralong GnRH agonist cycles. A subanalysis restricted to ultralong GnRHa cycles confirmed that the progesterone level is clearly superior to P/E2 in predicting clinical pregnancy. Because of the lack of large-scale research, controversy persists around this issue.

In our study, the number of follicles larger than 14 mm in diameter was larger in the high progesterone group, and the number of retrieved oocytes increased significantly. The results indicated that the increased progesterone level may be due to the excessive number of follicles. We noted that in the high progesterone group, the total dose of Gn was lower. However, a previous study [29] showed a strong positive correlation between the dose of FSH and the concentration of progesterone. This is contradictory to our results. The reason may be that the population we studied was PCOS patients. The characteristics of PCOS patients are heterogeneous. It can be seen from the results that the standard deviation of Gn dosage is very large, indicating that a large difference in the dosage of Gn used for different individuals exists. During the process of IVF/ICSI-ET, PCOS patients often show the characteristics of a high cycle cancellation rate, high ovarian response, poor oocyte quality, high incidence of ovarian hyperstimulation and more complications. Differences in gonadotrophin preparations used for ovarian stimulation may have differential effects on progesterone synthesis. In our study, although the number of obtained oocytes in the high progesterone group was larger, the number of high-quality embryos and implantation rate decreased. Moreover, the clinical pregnancy rate was not significantly changed, which suggested that although a high progesterone level can increase the number of oocytes, it may not have the capacity to improve the quality of eggs effectively. However, the clinical outcomes reported in this study are following the first embryo transfer and do not reflect cumulative pregnancy rates, where the total number of follicles (and thus oocytes retrieved and fertilized) may be more likely to play a role in predicting outcomes. In addition, we noted that women with fewer AFCs and mature follicles were more likely to end up pregnant. Presumably it’s not due to an egg quality issue but rather due to a higher rate of premature luteinization among the patients with higher AFCs, because these progesterone intermediates end up in sufficiently high circulating concentrations to impair the endometrial receptivity.

Interestingly, the number of oocytes obtained and rate of high-quality embryos in the high P/E2 group were decreased. The reason may be that the effect of the E2 level on oocyte development and maturation was greater than that of the progesterone level. It has been reported that E2 level is related to the number and size of follicles. To some extent, the E2 level represents the ovarian response to COS and indirectly reflects the quality of oocytes. Generally, with the increase in E2 level in blood, the size and number of follicles also increase. The increase in E2 can promote oocyte maturation by increasing meiotic ability [30]. In the present study, a high P/E2 ratio had no significant correlation with the embryo implantation rate, clinical pregnancy rate or live birth rate, and no significant difference was found in ectopic pregnancy rate and early miscarriage rate in all COS protocols. Multivariate analysis also indicated that the P/E2 ratio could not be used as a marker to predict the pregnancy outcome of PCOS patients. We observed that the implantation rate, hCG positive rate, clinical pregnancy rate and live birth rate were the highest in the ultralong GnRH agonist group, and they were statistically significant. Although a high P/E2 ratio on the hCG trigger day was found in the ultralong GnRH agonist, when subgroup analysis was performed, the P/E2 ratio had no correlation with the pregnancy outcome, suggesting that the P/E2 ratio in the ultralong GnRH agonist was not an independent factor affecting the pregnancy outcome. In our study, we noted that the patients with low P/E2 ratios are basically composed of patients with high E2 levels on average and progesterone levels comparable to those with high P/E2 ratios. Previous studies found that no association between single E2 concentrations and achievement of pregnancy [14]. This may be the reason why P/E2 ratio has no relationship with the outcome of pregnancy. ROC results indicated that the value of progesterone level in the prediction of clinical pregnancy was significantly higher than that of the P/E2 ratio. Combined assessment of the serum progesterone and P/E2 ratio may predict the pregnancy outcome better than progesterone levels alone [31]. During the COS process of the ultralong GnRH agonist protocol, pituitary gland function was fully downregulated, which effectively inhibited the endogenous LH peak and improved the reproductive internal environment of patients.

In the assisted reproductive technology cycle, overreaction to gonadotropin will lead to the risk of OHSS [32] and reduce the chance of pregnancy [33]. Patients with polycystic ovary syndrome are prone to OHSS. We observed that the number of AFCs in the ultralong GnRH agonist were significantly higher than those in the long GnRH agonist and GnRH antagonist. The cycle cancellation rate due to OHSS prevention increased significantly. A meta-analysis revealed that AMH and AFC are both accurate predictors of a high response, and both have clinical value [34]. In our center, AMH has only been tested in patients undergoing IVF/ICSI in recent years, thus, the AMH data could not be analyzed. However, the results of the high risk of OHSS in the high AFC group were consistent with previous studies.

Our study has the following limitations: First, because of the small sample size, this study failed to combine the progesterone level with the P/E2 ratio for analysis. Second, the clinical data of patients with FET cycles cannot be included in this study, thus, the results may not be sufficiently comprehensive. Third, in this study, we failed to analyze more clinical data of patients, such as the neonatal defect rate. Fourth, the current view is that oral contraception pretreatment can affect endometrial receptivity [35]. However, all patients in this study used oral contraceptive pretreatment, which may have an impact on the outcomes. Fifth, this study is a retrospective design. The choice of COS protocols depends on the clinicians and choice of patients. Thus, selective bias may exist. Our conclusion requires further confirmation in higher quality randomized controlled trials.

## Conclusion

Taken together, in PCOS patients, the progesterone level is associated with clinical pregnancy rate while P/E2 ratio is not. In subgroup analysis using three different COS protocols, a significant association between progesterone and clinical pregnancy rate can be observed in the long GnRH agonist protocol and ultralong GnRH agonist protocol. However, only in the ultralong GnRH agonist protocol is the progesterone level significantly more predictive than P/E2 ratio in prediction of clinical pregnancy.

## Supplementary Information

**Additional file 1.**

**Additional file 2.**

## Data Availability

The datasets used and/or analysed during the current study are available from the corresponding author on reasonable request.

## Abbreviations

PCOS: Polycystic ovary syndrome
BMI: Body mass index
FSH: Follicle-stimulating hormone
E2: Estradiol
LH: Luteinizing hormone
P: Progesterone
Gn: Gonadotropin
hCG: Human chorionic gonadotropin
IVF: In vitro fertilization
ART: Assisted reproductive technology
GnRH: Gonadotropin-releasing hormone
ICSI: Intracytoplasmic sperm injection
ET: Embryo transfer
PGD: Preimplantation genetic diagnosis
PGT: Preimplantation genetic testing
AFC: Antral follicle count
COS: Controlled ovarian stimulation
AUC: Area under the curve
